# Discovery of novel frizzled-7 inhibitors by targeting the receptor’s transmembrane domain

**DOI:** 10.18632/oncotarget.20665

**Published:** 2017-09-06

**Authors:** Wei Zhang, Wenyan Lu, Subramaniam Ananthan, Mark J. Suto, Yonghe Li

**Affiliations:** ^1^ Department of Chemistry, Drug Discovery Division, Southern Research Institute, Birmingham, Alabama 35205, United States; ^2^ Department of Oncology, Drug Discovery Division, Southern Research Institute, Birmingham, Alabama 35205, United States

**Keywords:** frizzled, Wnt/β-catenin, inhibitor, virtual screening, cancer

## Abstract

Frizzled (Fzd) proteins are seven transmembrane receptors that belong to a novel and separated family of G-protein-coupled receptors (GPCRs). The Fzd receptors can respond to Wnt proteins to activate the canonical β-catenin pathway which is important for both initiation and progression of cancers. Disruption of the Wnt/β-catenin signal thus represents an opportunity for rational cancer prevention and therapy. Of the 10 members of the Fzd family, Fzd7 is the most important member involved in cancer development and progression. In the present studies, we applied structure-based virtual screening targeting the transmembrane domain (TMD) of Fzd7 to select compounds that could potentially bind to the Fzd7-TMD and block the Wnt/Fzd7 signaling and further evaluated them in biological assays. Six small molecule compounds were confirmed as Fzd7 inhibitors. The best hit, SRI37892, significantly blocked the Wnt/Fzd7 signaling with IC_50_ values in the sub-micromolar range and inhibited cancer cell proliferation with IC_50_ values around 2 μM. Our results provide the first proof of concept of targeting Fzd-TMD for the development of Wnt/Fzd modulators. The identified small molecular Fzd7 inhibitors can serve as a useful tool for studying the regulation mechanism(s) of Wnt/Fzd7 signaling as well as a starting point for the development of cancer therapeutic agents.

## INTRODUCTION

Frizzled (Fzd) proteins are a family of seven transmembrane receptors (7-TM) for secreted Wnt glycolipoproteins. The Fzd receptors can respond to Wnt proteins in the presence of the Wnt co-receptor low density lipoprotein receptor-related protein 5 (LRP5) or LRP6 to activate the canonical β-catenin pathway. Activation of Wnt/β-catenin signaling has been found to be important for both initiation and progression of cancers of different tissues [1, 2]. Thus, disruption of the Wnt/β-catenin signal represents an opportunity for rational cancer chemoprevention and therapy [3, 4, 5].

Of the 10 members of the Fzd family, Fzd7 is the most important member regulating cancer development and progression. Fzd7 is frequently up-regulated in a variety of cancers including colon cancer [6, 7], breast cancer [8], hepatocellular carcinoma [9, 10], lymphoblastic leukemia [11], Wilm’s tumors [12, 13], glioblastoma [14], ovarian cancer [15] melanoma [16], chronic myeloid leukemia [17] and gastric cancer [18]. Recent studies have demonstrated that the anti-Fzd7 antibody [12, 19, 20, 21], siRNA/shRNA knockdown of Fzd7, [6, 7, 12, 15, 16, 17], soluble Fzd7 peptides [22] and small interfering peptides directed against Fzd7 [23] antagonized Wnt/β-catenin signaling in Fzd7-expressing cancer cells, and suppressed tumor progression and metastasis. Therefore, targeted inhibition of Fzd7 represents a rational and promising new approach for cancer therapy [24, 25].

The Fzd family (Fzd_1-10_) and the closely related Smoothened (SMO) have been listed by the International Union of Pharmacology (IUPHAR) as a novel and separated family of G-protein-coupled receptors (GPCRs) : the class-F (Frizzled) [26]. In addition to the traditional seven transmembrane helices domain (7TMD), Fzds also process a large N-terminal extracellular cystein rich domain (CRD) and a C-terminal intracellular PDZ (Psd-95/disc large/ZO-1 homologs)-binding domain. The CRD is fully exposed and interacts with extracellular Fzd binding proteins, including the receptor’s cognate ligands Wnt proteins [27, 28]. The PDZ-binding domain interact with the intracellular Fzd binding proteins, including Disheveled (Dvl), an essential and functionally necessary phosphoprotein for Fzd signaling pathways [29, 30]. While it has been demonstrated that interruption of the extracellular Wnt/CRD [19, 31], or the intracellular Fzd/Dvl [32, 33] interactions could effectively inhibit Wnt/β-catenin signaling, studies targeting the TMD of Fzd have not been reported to date.

The TMD of Fzd shares the same topology as classic GPCRs, including the conventional 7TM core, the intracellular C-terminal helix-8, the conserved cysteine residues in the extracellular loops as well as other common residues in the TMD of GPCRs [34, 35]. The genuine GPCR features of Fzd, including both G-protein and β-arrestin dependent signaling pathways, have also been observed in recent years [36, 37, 38, 39]. As major targets for drug development, GPCRs have been studied for many years and it is now well accepted that GPCR proteins exist in a conformational equilibrium between active and inactive biophysical states [40, 41, 42; 43], and ligands that shift the equilibrium toward the active/inactive conformational states of GPCRs have had applications in many different therapeutic areas. Therefore, it would be interesting to find out whether targeting Fzd-TMD could be an alternative strategy to modulate the Wnt/Fzd signaling.

The recently reported crystal structure of the TMD of human SMO receptor bound to a small-molecule antagonist [44] further supports the notion of blocking Wnt signaling using small molecule inhibitors. It also provides an excellent structural template for molecular modeling study of Fzd-TMD. In the present study, we constructed a homology model of the TMD of Fzd7 and applied structure-based virtual screening (SBVS) to select putative small molecular Fzd7 inhibitors. Our study led to the identification of an Fzd7 inhibitor, SRI37892, with potent activities against Wnt/β-catenin signaling and cancer cell viability.

## RESULTS

### Homology modeling and structural analysis of Fzd7-TMD

**Figure 1:**
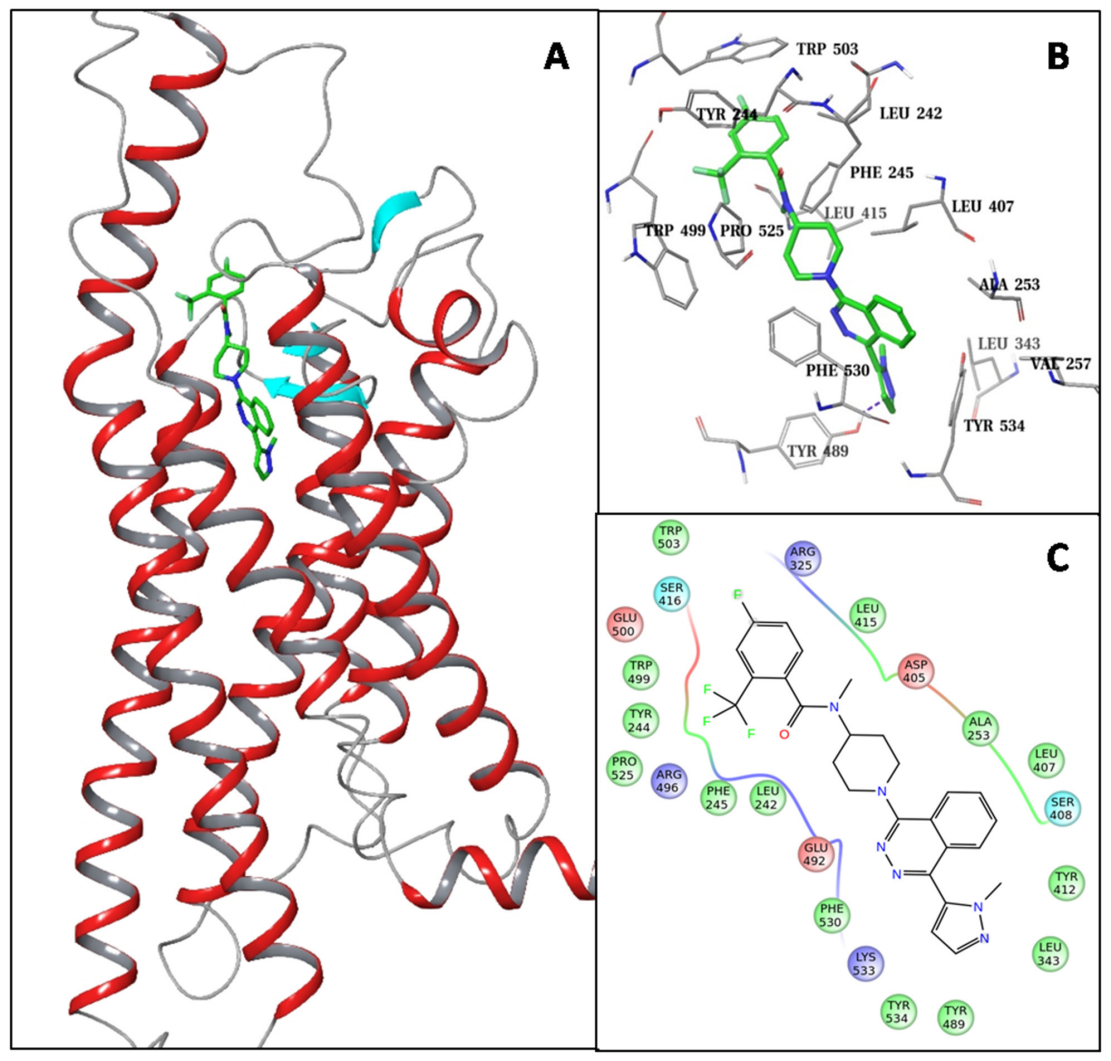
Structural presentation of the homology model of Fzd7-TMD **(A)** Ribbon presentation of the Fzd-TMD model with LY2940680 (shown in solid sticks) buried in the putative binding site. **(B)** Close-up view of the binding site residues that form the top and the bottom hydrophobic cores. **(C)** 2D interaction diagram showing all binding site residues. Residues are colored by properties: hydrophobic (green), polar (cyan), positively charged (blue), negatively charged (red).

The SMO receptor has high sequence similarity to the frizzled receptors. The TMDs of Fzd7 and SMO share 28% identical and 47% homologous residues. Based on the SMO crystal structural and multi-sequence alignment of the GPCR class-F family (see Supplementary Tables 1 and 2 in the supporting information), we constructed a homology model for the Fzd7-TMD (Figure 1A). The SMO antagonist, LY2940680, which was co-crystallized in the SMO crystal structure was kept in the Fzd7-TMD model as a reference molecule to hold the putative ligand binding site. The top part of this putative binding site of the Fzd7 model is exposed and surrounded by the extracellular loops (ELs) as well as the N-terminal linker region (NLR), the rest part of the binding site is buried in the TMD and formed by residues from transmembrane helices (THs) I, III, VI and VII. This binding site mainly consists of hydrophobic residues which form two hydrophobic cores at the top and the bottom regions of the pocket (Figure 1B and 1C). The top hydrophobic core is formed by residues from NLR (L242, Y244, F245), EL-2 (L407, L415) and EL-3 (W499, W503, P525), the bottom core includes residues from TH-I (A253^1.35^, Y257^1.39^), TH-III (L343^3.32^), TH-VI (Y489^6.52^), TH-VII (F530^7.38^, F534^7.42^) as well as a residue Y412 from EL-2. Results of multiple sequence alignment of the class-Frizzled showed that the properties of most of the binding site residues are well conserved (see Supplementary Table 1 in the supporting information), especially residues that form the two hydrophobic cores, which suggests that this putative ligand binding site may exist commonly in the Frizzled family. These results encouraged us to target this binding site of Fzd7-TMD for small molecule inhibitors.

### Identification of Fzd7 inhibitors through SBVS

**Figure 2:**
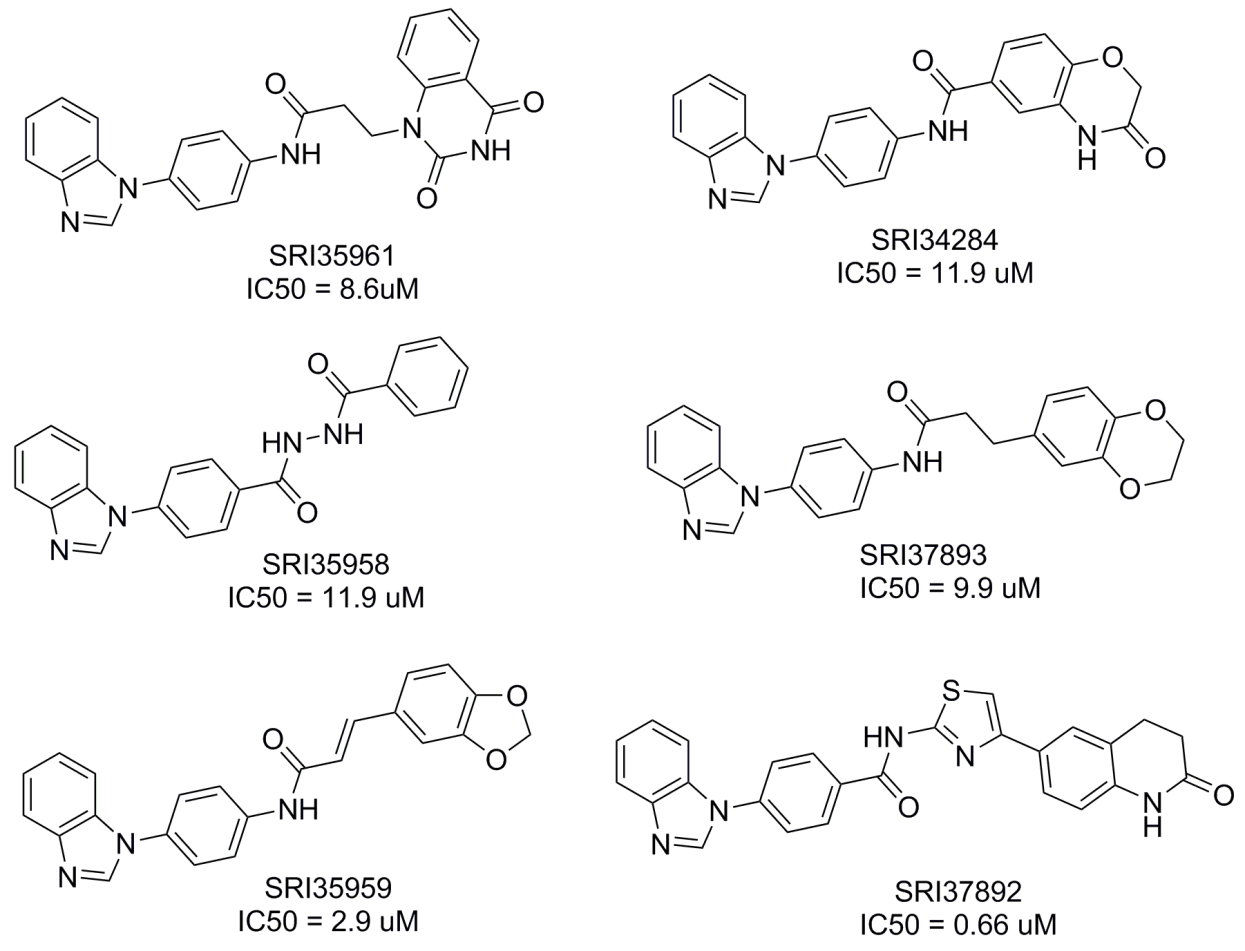
Structures of the identified small molecule Fzd7 inhibitors The IC_50_ values for inhibition of Wnt/β-catenin signaling were measured in Wnt3A-expressing HEK293 cells as described in Figure 3A and 3C.

**Figure 3:**
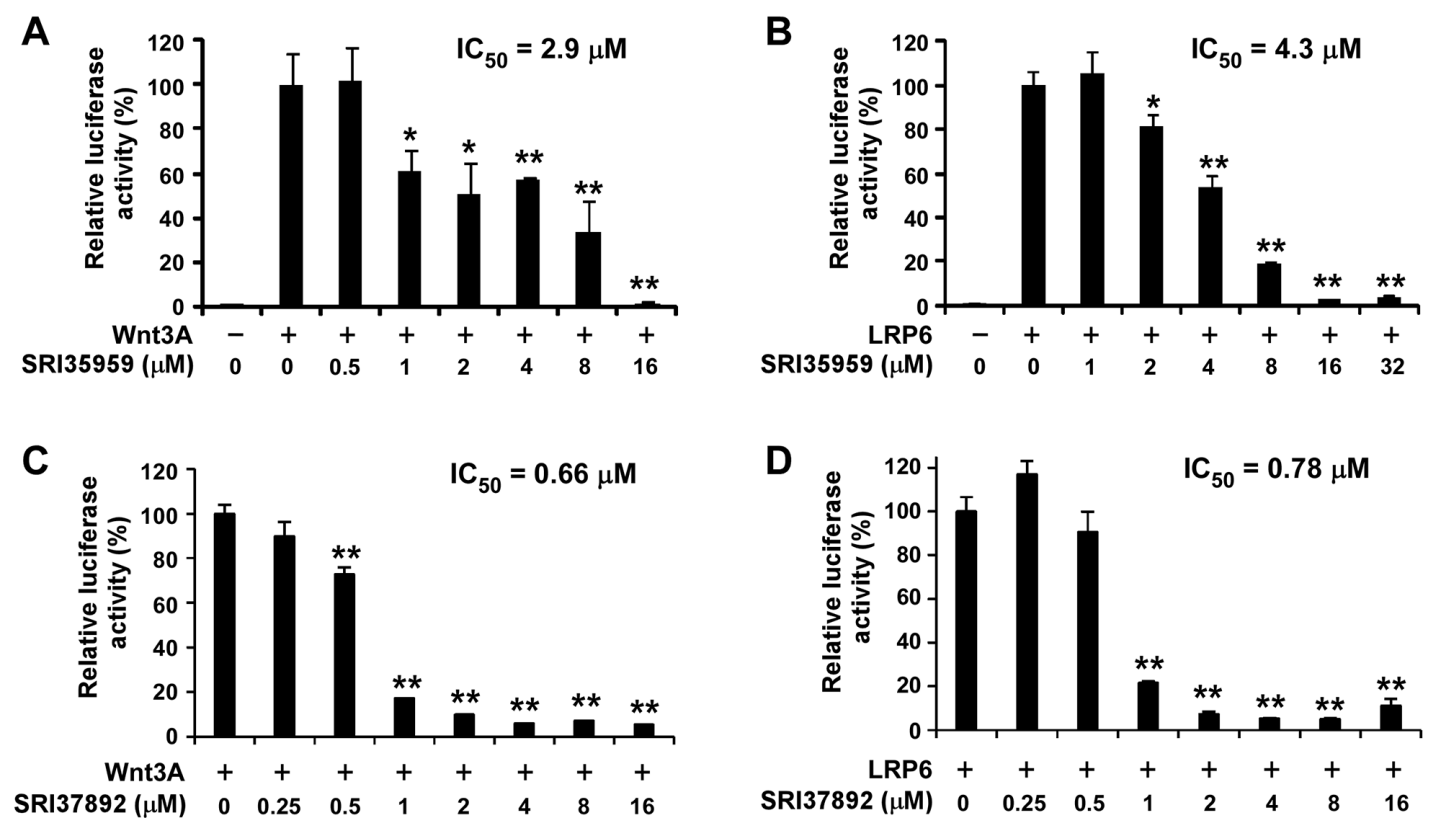
SRI35959 and SRI37892 block Wnt/β-catenin signaling in HEK293 cells induced by Wnt3A and LRP6 HEK293 cells in 24-well plates were transiently transfected with the Wnt3A plasmid **(A, C)** or the LRP6 plasmid **(B, D)** along with the Super8XTOPFlash luciferase construct and the β-galactosidase-expressing vector in each well. After 24 h incubation, cells were treated with SRI35959 (A, B) or SRI37892 (C, D) at the indicated concentrations for 24 h. The luciferase activity was then measured with normalization to the activity of the β-galactosidase. Values are the average of triple determinations with the SD indicated by error bars. *P < 0.05, **P < 0.01 versus corresponding control value.

To identify compounds that can bind to the Fzd7-TMD and block Wnt/Fzd7 signaling, we conducted structure based virtual screening (SBVS) targeting the TMD binding site of the constructed Fzd7 model. 500,000 structurally diverse compounds were screened through a three-step docking/scoring process using the Glide program. The docked results of 738 top-scored compounds at the final docking stage were further visually inspected to select potential Fzd7 inhibitors that showed both shape and electrostatic complementarities to the receptor binding site. We hypothesized that occupying the two hydrophobic cores could be important for ligand binding and gave certain priorities to compounds that can fit into both hydrophobic regions, especially to those that fitted well to the bottom hydrophobic core. By taking into consideration structural diversity in our final selection, 67 structurally representative compounds were purchased and tested for their inhibitory effect on Wnt/β-catenin signaling in Wnt3-expressing HEK293 cells. At 10 μM concentrations, 3 of 67 compounds, SRI35959, SRI35961 and SRI35958, significantly ( > 50%) blocked Wnt3A induced Wnt/β-catenin signaling and their inhibitory effects were further confirmed in concentration dependent assays with IC_50_ values of 2.9, 8.6 and 11.9 μM, respectively (Figures 2 and 3A). SRI35959, the most potent of these three hits, was further tested in LRP6-expressing HEK293 cells, and was found to display a similar inhibition potency with an IC_50_ value of 4.3 μM (Figure 3B).

While the 67 selected compounds included structurally diverse compounds (see Supplementary Table 3 in the supporting information), the three hits identified clearly share some structural similarities, including the presence of a common phenylbenzimidazole unit. The most potent compound, SRI35959, however, possesses a potentially reactive α,β-unsaturated amide group and a 1,3-benzdioxole moiety that is prone for metabolic conversion to toxic metabolites [45]. Therefore, to identify additional compounds with improved potency and devoid of structural liabilities, we conducted analog searching and docking-screening to select a second set of compounds. By using different analog search methods, including similarity, substructure and topomer searches, we assembled 5000 “analogs” and docked them into the binding site of our Fzd7-TMD model. Following the same compound selection procedures described above, 35 analogs (Supplementary Table 4) were finally purchased from the top-scored results and tested in the Wnt/β-catenin assay. Three of 35 compounds, SRI37892, SRI37893 and SRI34284, were confirmed as actives with IC_50_ values of 0.66, 9.9 and 11.9 μM, respectively (Figures 2 and 3C). The best hit, SRI37892, also displayed potent activity against Wnt/β-catenin signaling in LRP6-expressing HEK293 cells with an IC_50_ value of 0.78 μM (Figure 3D).

All the 6 hits from the two sets of tested compounds share the same phenylbenzimidazole fragment, suggesting this group may be important for receptor binding. 4 of the 6 hits (SRI35959, SRI35961, SRI37893 and SRI34284) possess a potentially metabolically oxidizable p-phenylenediamine-like unit. The most potent compound, SR37892, however, does not contain unfavorable chemical features and was therefore selected for further studies described below.

### Inhibitory effects of SRI37892 on Wnt/β-catenin signaling in cancer cells

**Figure 4:**
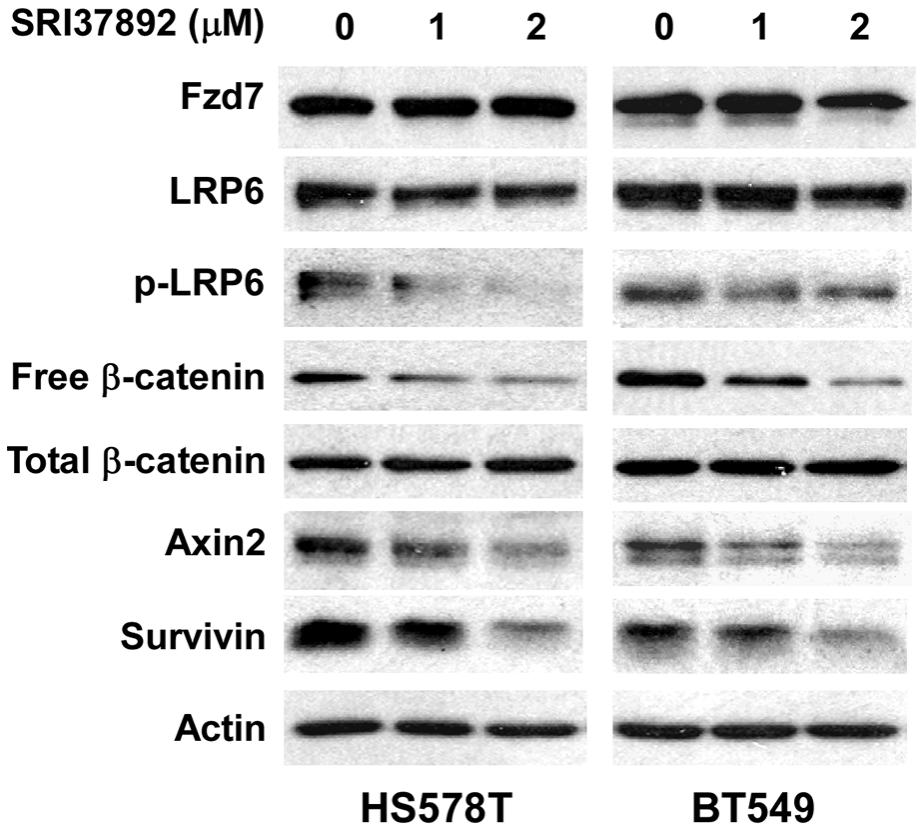
Effects of SRI37892 on Wnt/β-catenin signaling in breast cancer HS578T and BT549 cells Cancer cells in 6-well plates were treated with SRI37892 at the indicated concentrations for 24 h. The levels of cytosolic free β-catenin, total cellular β-catenin, Fzd7, LRP6, phospho-LRP6, axin2 and survivin were examined by Western blotting. All the samples were also probed with anti-human actin antibody to verify equal loading.

**Figure 5:**
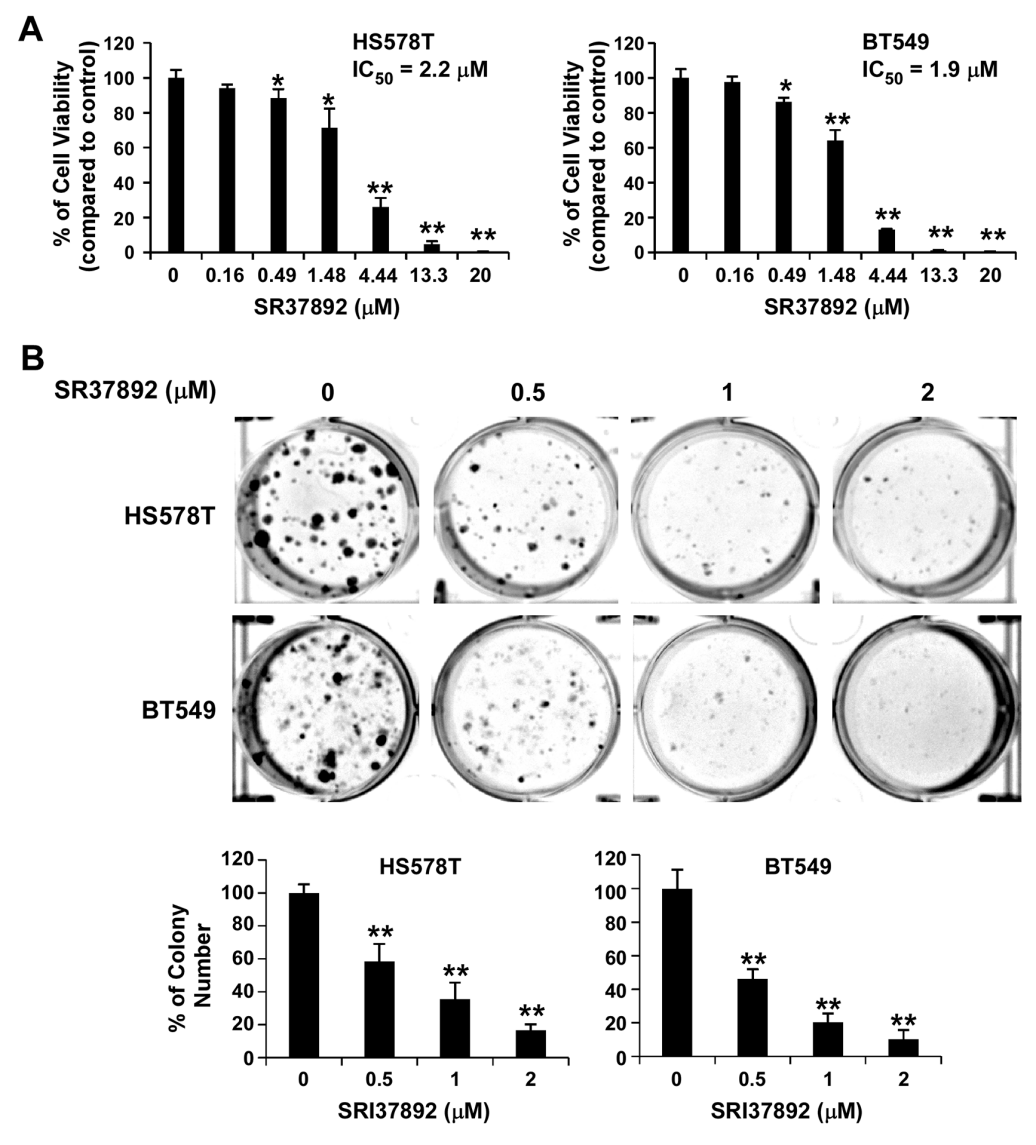
SRI37892 inhibits breast cancer cell viability and colony formation **(A)** Breast cancer HS578T and BT549 cells in 96-well plates were treated with SRI37892 at the indicated concentrations for 96 h. Cell viability was measured by the CellTiter-Glo assay. **(B)** Breast cancer HS578T and BT549 cells were treated with SR37892 at the indicated concentrations for 10–12 days. The medium was changed every 3 days. Colonies were fixed with formaldehyde and stained with Crystal Violet. All values are averages of triplicate determinations with the S.D. indicated by error bars. **P <0.01 compared with corresponding control value.

Aberrant Wnt/β-catenin signaling is associated with a poorer prognosis in breast cancer patients [46], and is predominantly found in triple negative breast cancer (TNBC) which is distinguished by negative immunohistochemical assays for expression of the estrogen and progesterone receptors (ER/PR) and human epidermal growth factor receptor-2 (HER2) [47, 48, 49]. It has been reported that FZD7 is upregulated in TNBC, and that FZD7 plays an important role on Wnt/β-catenin signaling in TNBC cells and cancer cell proliferation [50]. Therefore, we tested SRI37892 in TNBC HS578T and BT549 cells to confirm its inhibitory effect on Wnt/β-catenin signaling. As expected, treatment of SRI37892 at 1 or 2 μM resulted in suppression of LRP6 phosphorylation, down-regulation of cytosolic free β-catenin level, and inhibition of expression of specific Wnt targets axin2 and survivin in HS578T and BT549 cells (Figure 4). Moreover, SRI37892 displayed potent activity against HS578T and BT549 cell proliferation with IC_50_ values of 2.2 and 1.9 μM, respectively (Figure 5A). Finally, SRI37892 at 0.5-2 μM significantly suppressed colony formation in HS578T and BT549 cells (Figure 5B).

### Predicted binding mode of the active compounds

**Figure 6:**
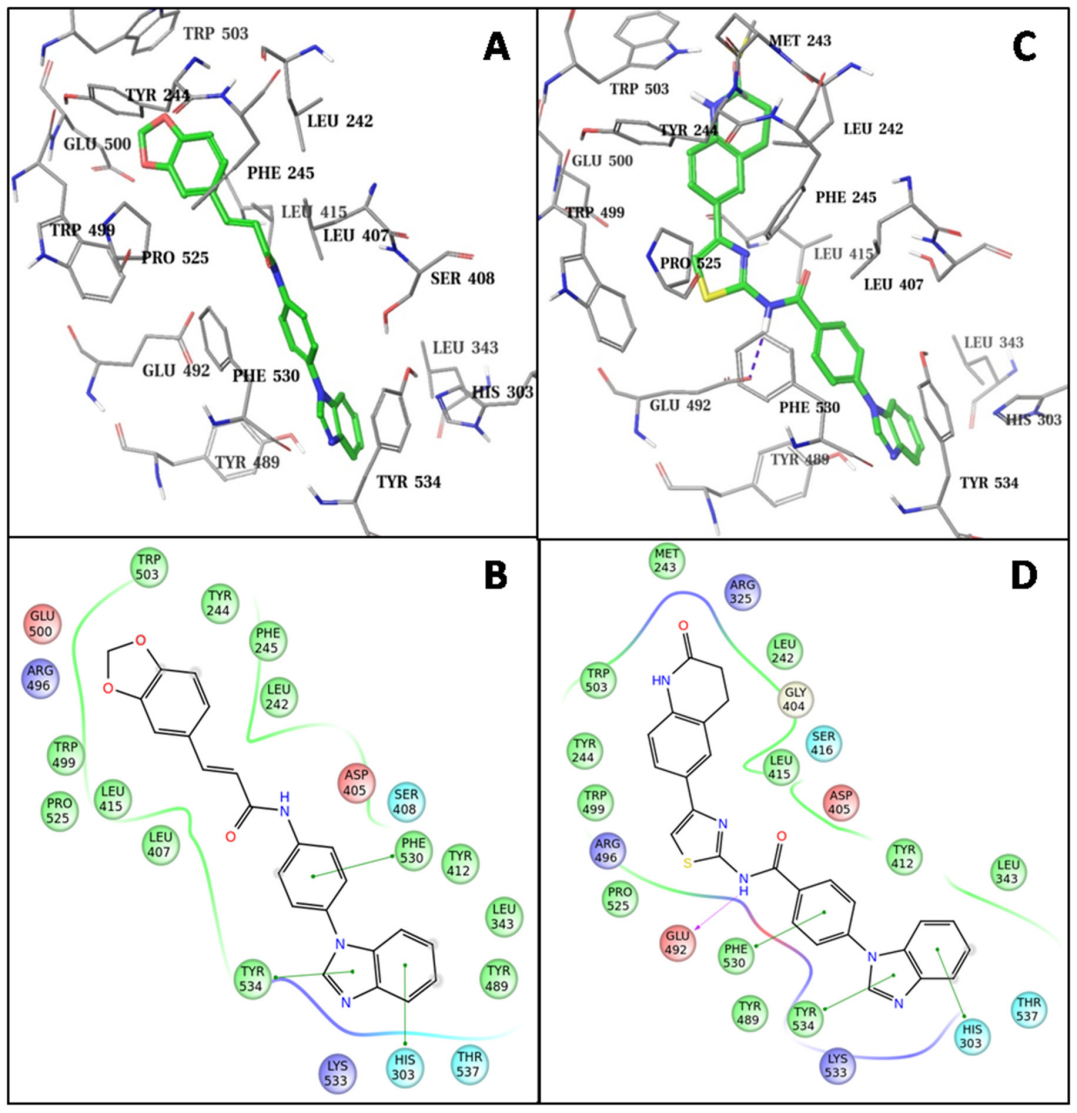
Predicted binding modes of identified Fzd7 inhibitors The docked models of SRI35953 **(A)** and SRI37892 **(B)**. Inhibitor molecules are represented in green-colored solid sticks. Protein residues are shown in gray lines. 2D ligand interaction diagram of SRI35953 **(C)** and SRI37892 **(D)**. Pi-Pi stacking is marked with solid green lines. Hydrogen bond is marked with dashed purple lines. Residues are colored by properties: hydrophobic (green), polar (cyan), positively charged (blue), negatively charged (red).

To gain structural insights into Fzd7-inhibitor binding, we further conducted docking studies following a more sophisticated induced-fit docking (IFD) protocol which allows binding site residues to be flexible. The two most potent hits, SRI35959 and SRI37892, were docked separately into the putative binding site of the Fzd7-TMD model. The docked models suggested that SRI37892 bound relatively tighter than SRI35959 with a docking score (which mimics the binding affinity) of -12.0 kcal/mol as compared to -10.3 kcal/mol of SRI35959, which is consistent with the observed experimental results that SRI37892 is a more potent inhibitor. Both compounds fit well into the Fzd7 binding site. In the docked models (Figure 6), the phenylbenzimidazole moiety of these two compounds docked very similarly: both occupied the lower hydrophobic pocket of the binding site and formed multiple Pi-Pi interactions with the aromatic sidechains of the surrounding residues (Trp534, Phe530 and His303). While the other end of the two compounds occupied the top hydrophobic core of the binding site, there are clear differences among the protein-inhibitor interactions. SRI35959 has a relatively linear conformation and thus its benzodioxole end can only occupy part of the top hydrophobic core. In contrast, the kinked conformation of SRI37892 enables its dihydroquinolinone end to interact with residues at the other side of the top hydrophobic core, including Leu242 and Met243 from the N-terminal linker region. While the middle fragment of SRI35959 did not form any close interactions with the receptor, the amide group of SRI37982 formed a hydrogen bond with Glu492 and its middle thiazole ring fitted tightly into a hydrophobic region formed by Pro525 and Trp499. Taken together, occupying both the top and the bottom hydrophobic regions of the binding site is likely important for ligand binding, and forming close hydrophobic interactions and additional hydrogen bond(s) with nearby residues can further contribute to favorable Fzd7-inhibitor interactions.

## DISCUSSION

In addition to the canonical β-catenin dependent signaling, there are also other noncannonical Wnt/Fzd signaling pathways that are independent of β-catenin, including Wnt/Ca^2+^ and Wnt/PCP signaling [51]. The mechanism(s) that controls the activation of a Wnt toward a certain signaling path are still unclear. Meanwhile, while structure biology studies have demonstrated that Wnts bind directly to the extracellular CRD of Fzd [52], other studies suggested that the CRD might be dispensable for the Wnt/Fzd signaling [53]. A possible explanation for such a conundrum is that CRD may act as a ligand fishing rod that brings Wnt close to the receptor thus facilitating further interactions with other regions of the core TMD to activate the Wnt/Fzd signaling [54]. Nonetheless, the underlying mechanism(s) of Fzds modulated signaling is still unclear partly because of the lack of proper research tools. To the best of our knowledge, this is the first report on small molecule Wnt inhibitors that target the Fzd-TMD. The identified Fzd7 inhibitors thus provide a useful tool for the study of Wnt/Fzd signaling mechanism.

Wnt/β-catenin signaling is activated aberrantly in many human cancers, and FZD7 is frequently up-regulated in a variety of cancers including breast and colorectal cancer [24, 25]. While genetic mutations of certain components of the Wnt/β-catenin pathway are not the predominate mechanism associated with many types of cancer including breast cancer [4], mutations of *APC* and *CTNNB1* (β-catenin encoding gene) are two major factors for Wnt/β-catenin signaling overactivation in colorectal cancers [55, 56]. However recent studies also indicate that the levels of Wnt/β-catenin signaling in colorectal cancer cells can be modulated on the cell surface [57, 58, 59]. Fzd7 can activate Wnt/β-catenin signaling in colorectal cancer cells despite the presence of *APC* or *CTNNB1* mutations [6, 7]. In the present study, we demonstrated that SRI37892 has a potent activity against Wnt/β-catenin signaling in TNBC cells. It would be interesting to see whether SRI37892 can indeed effectively inhibit Wnt/β-catenin signaling in colorectal cancer cells harboring *APC* and *CTNNB1* mutations.

Wnt/β-catenin signaling is a critical regulator of stem cells [2]. Recent studies have demonstrated that Fzd7 is required for maintenance of the pluripotent state in human embryonic stem cells [60], and functions as a Wnt receptor for robust Wnt-dependent processes in Lgr5(+) intestinal stem cells [61]. Moreover, Fzd7 plays an important role in activation of the non-canonical Wnt signaling pathway during human embryonic hematopoiesis [62]. Further studies are required to examine whether the identified compounds inhibit non-canonical Wnt signaling and modulate stem cell fate and function.

While increased Wnts expression has been reported in different forms of cancers, reduced expression of Wnts is associated with other disorders such as obesity [63]. Recent studies also demonstrated that the Wnt/β-catenin pathway is a critical regulator of osteoclastogenesis, and that Wnt activation attenuates osteoclast differentiation and bone resorption, which suggests that activating Wnt/β-catenin signaling is an attractive strategy for the treatment of bone loss disorders [64, 65, 66]. Targeting the TMD of GPCRs is a clinically validated strategy for drug development. Ligands that bind to the TMD and modulate the conformations of GPCRs have been successfully applied to develop different drug candidates, including agonists, antagonist as well as biased modulators that regulate specific signaling pathways [67, 68, 69, 70, 71]. The GPCR characterizations of Fzds have been established in recent years. Therefore, targeting Fzd-TMD could be an attractive therapeutic strategy to modulate Wnt/Fzd signaling. Nonetheless, so far there were no studies demonstrating the feasibility of such a strategy. In the present study, by specifically targeting a putative binding site at the Fzd7-TMD, we successfully identified small molecule compounds that significantly blocked the Wnt/Fzd signaling and inhibited the proliferation of cancer cells, thus providing a strong support to the concept of targeting Fzd-TMD for the development of chemotherapeutic candidates. Sequence analysis shows that most of the SRI37892 binding site residues of Fzd7-TMD are well conserved (see Supplementary Tables 1 and 2 in the supporting information). Therefore, the compounds identified here may also bind to other FZD receptors. Nevertheless, the identified SRI37892, a potent Fzd7 inhibitor, represents a potential lead for further drug development.

## MATERIALS AND METHODS

### Molecular modeling

Structural model generation and molecular docking studies were conducted using the programs of the Schrödinger Suite 2015 (*Schrödinger, LLC, New York, NY, 2015*).

Homology modeling was conducted using the *Prime* program. The crystal structure of SMO-LY2940680 (a small-molecule SMO antagonist) complex (PDB ID: 4JVK) was used as the structural template. The sequences of SMO and frizzled receptors (Fzds_1-10_) was first aligned using *ClusterX*. The alignment results were then input into *Prime* to build the structural model of Fzd7-TMD. We also used the Ballesteros and Weinstein numbering system [72] on the basis of structural superposition. Specifically, for each of the THs, the following residues are assigned number 50: T268^1.50^, Y296^2.50^, W361^3.50^, W386^4.50^, Y432^5.50^, A487^6.50^, I542^7.50^.

Molecular docking studies were performed using the *Glide* program with the default parameters. The 3D structures of ligands were prepared using the *LigPrep* program. The induced-Fit-Docking (IFD) protocol [73], which is capable of sampling dramatic side-chain conformational changes as well as minor changes in protein backbone structure, was applied to explore the potential binding modes of identified active compounds. Residues within 5 Å of the docked ligands were allowed to be flexible and the docked results were finally scored using the extra-precision (XP) mode of *Glide*.

### Structure based virtual screening

For the purpose of applying structure-based virtual screening, we assembled a virtual library consisting structurally diverse compounds selected from different commercial sources. Specifically, structures of a total of approximately eight million compounds were downloaded directly from the websites of twelve vendors (Asinex, Chembridge, ChemDiv, Enamine, FCH group, InterBioScreen, Life chemicals, Maybridge, Princeton, TIMTEC, SPECS and Vitas-M). From the eight million commercially available compounds, we first identified the most diverse set of 100,000 structurally representative compounds using the clustering and diversity analysis protocols of *Pipeline Pilot*; [74] then for each of the 100,000 compounds, we further selected four structurally most similar analogs from the rest of the eight million compounds based on the Tanimoto coefficients calculated from the 2D structural fingerprints. Such an assembled library of 500,000 compounds covers a large portion of chemical space, contains diverse structures, and could provide initial structure-activity relationship (SAR) information.

Structure-based virtual screening was performed using the virtual screening workflow of *maestro,* which adopts a three-step docking/scoring protocol implemented in *Glide*. 3D conformations of the 500,000 assembled compounds were prepared using *LigPrep,* and a total of 923,408 conformers were generated. All of the 923,408 conformers were first docked into the ligand binding site of the Fzd7-TMD model using the high-throughput virtual screening (HTVS) mode; the top 5% best-scored conformers were then re-docked and scored using the standard-precision (SP) mode; the top 10% of the SP docked conformers were further docked and scored using the extra-precision (XP) mode. Finally, the top 25% of the best XP-scored conformers were output for visual examination.

### Analog search

Commercial analogs of confirmed active compounds were identified through substructure search and similarity search protocols of *Pipeline Pilot* and the TOPOMER search module of *SYBYLX* program. The identified analogs were then docked to the binding site of the Fzd7-TMD model. The top-scored docked models were further visually examined for structural complementary in both shape and polarity to the receptor to select a final list of compounds for biological evaluations.

### Cell culture

All cell lines were obtained from ATCC and grown under standard cell culture conditions at 37°C in a humidified atmosphere with 5% CO2. The cells were cultured in DMEM medium containing 10% of FBS, 2 mM of L-glutamine, 100 units/ml of penicillin, and 100 μg/ml of streptomycin.

### Luciferase Wnt reporter assay

Plasmid pCS-Myc-hLRP6 containing the full-length human LRP6 cDNA was provided by Dr. Christof Niehrs (Deutsches Krebsforschungszentrum, Heidelberg, Germany), and the Super8XTOPFlash luciferase construct was provided by Dr. Randall T. Moon (University of Washington, Seattle). Plasmid pcDNA3-Wnt3-HA was constructed as described previously [75]. HEK293 cells were plated into 24-well plates. After overnight culture, the cells were transiently transfected with the Super8XTOPFlash luciferase construct and β-galactosidase-expressing vector (Promega) along with or without Wnt3A or LRP6 plasmid. After 24 h incubation, cells were treated with the test compounds at the indicated concentrations. Cells were then lysed 24 h later and both luciferase and β-galactosidase activities were determined. The luciferase activity was normalized to the β-galactosidase activity.

### Western blotting

Polyclonal anti-LRP6 and monoclonal anti-survivin were from Santa Cruz Biotechnology. Polyclonal anti-Fzd7 was from Novus Biologicals. Monoclonal anti-phospho-LRP6 and anti-axin2 were purchased from Cell Signaling Technology. Monoclonal anti-β-catenin was from BD Biosciences. Monoclonal anti-actin was from Sigma. Cells in 6-well plates were lysed in 0.5 ml of lysis buffer (phosphate-buffered saline containing 1% Triton X-100 and 1 mM phenylmethylsulfonyl fluoride) at 4°C for 30 min. Equal quantities of protein were subjected to SDS-PAGE under reducing conditions. Following transfer to immobilon-P transfer membrane, successive incubations with a primary antibody, and a horseradish peroxidase-conjugated secondary antibody were carried out for 60-120 min at room temperature. The immunoreactive proteins were then detected using the ECL system. Films showing immunoreactive bands were scanned by HP Scanjet 5590 (Hewlett Packard, Palo Alto, CA).

### Cytosolic free β-catenin analysis

Plasmid pGST-E-cadherin was provided by Dr. Gail Johnson (University of Rochester). The level of cytosolic free β-catenin was analyzed by the GST-E-cadherin binding assay as previously described [75]. Uncomplexed cytosolic free β-catenin present in 100 μg of total cell lysate was subjected to SDS-PAGE and detected using the monoclonal antibody to β-catenin (BD Biosciences).

### Cell viability assay

Cells were seeded into 96-well tissue culture treated microtiter plates at a density of 4000 cells/well. After overnight incubation, the cells were treated with each individual compound for 96 h. Cell viability was measured by the CellTiter-Glo Assay (Promega).

### Colony formation assay

Cancer cells were seeded at a density of 800 cells/well into six-well plates. Sixteen hours after the plates had been set up, SR37892 was added, and media were replenished every 3 days. After being incubated for 12 days, colonies were fixed with 4% formaldehyde, stained with 0.5 mg/ml crystal violet, and imaged on a FluorChem HD2 Imager System (Alpha Innotech).

## SUPPLEMENTARY MATERIALS TABLES








