# Supplementary material for: Discovery of novel frizzled-7 inhibitors by targeting the receptor’s transmembrane domain
Source: Oncotarget. 2017 Sep 6;8(53):91459–70. doi: 10.18632/oncotarget.20665 (PMC5710937; doi:10.18632/oncotarget.20665)
Supplement: Supplementary file 1 [file oncotarget-08-91459-s001.pdf]

Discovery of novel frizzled-7 inhibitors by targeting the receptor's transmembrane domain

SUPPLEMENTARY MATERIALS

Supplementary Table 1: Sequence alignment of the binding site residues. ***NLR**: N-terminal linker region; **TH**: transmembrane helix; **EL**: extracellular loop. **Residues are colored based on their properties: hydrophobic-green; polar-cyan; positively charged-red; negatively charged-blue.

See Supplementary File 1

Supplementary Table 2: Result of multiple sequence alignment of the frizzled family*

```
FZD7_      ---------------------------------------------------------------------MRDPGAAAPLSSLGLCALVLALLGALSAGAG    31
FZD1_      -MAEEEAPKKSRAAGGGASWELCAGALSARLAEEGSGDAGGRRRPPVDPRRLARQLLLLLWLLEAPLLLGVRAQAAGQGPGQGPGPGQQPPPPPQQQQSG    99
FZD2_      ARGVSGCSVRPRQASGQAHRPLPRPASPTLRPRVKFAKRRGRRQPQRGGGGEEAQ--SPGWGRGRGGRQGAGWGAAASMRPRSALPRLLLPLLLLPAAGP    98
FZD3_      ------------------------------------------------------------------------------MAMTWIVFSLWPLTVFMGHIGG    22
FZD4_      --------------------------------------------------------------------MAWRGAGPSVPGAPGGVGLSLGLLLQLLLLLG    32
FZD5_      ----------------------------------------------------------------------------MARPDPSAPPSLL--LLLLAQLVG    22
FZD6_      ----------------------------------------------------------------------------------MEMFTFLLTCIFLPLLRG    18
FZD8_      ----------------------------------------------------------------------------MEWGYLLEVTSLLAALALLQRSSG    24
FZD9_      --------------------------------------------------------------------MAV--APLRGALLLWQLLAAGGAALEIGRFD-    30
FZD10_     --------------------------------------------------------------------------MQRPGPRLWLVLQVMGSCAAISSMDM    26
SMO_       -----------------------------------------MAAARPARGPELPLLGLLLLLLLGDPGRGAASSGNATGPGPRSAGGSARRSAAVTGPPP    59
           1.......10........20........30........40........50........60........70........80........90.......100
```

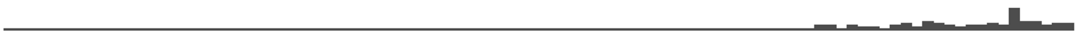

```
                            *: :    *    : *. *      .   *  *   :  :  : :   *   :. :**  : * *          .  :*  .
FZD7_      AQPYHGEKGISVPDHGFCQPISIPLCTD--IAYNQTILPNLLGHTNQEDAGLEVHQFYPLVKVQ-CSPELRFFLCSMYAPVCT-VLDQAIPPCRSLCERA    127
FZD1_      QQ-YNGERGISVPDHGYCQPISIPLCTD--IAYNQTIMPNLLGHTNQEDAGLEVHQFYPLVKVQ-CSAELKFFLCSMYAPVCT-VLEQALPPCRSLCERA    194
FZD2_      AQ-FHGEKGISIPDHGFCQPISIPLCTD--IAYNQTIMPNLLGHTNQEDAGLEVHQFYPLVKVQ-CSPELRFFLCSMYAPVCT-VLEQAIPPCRSICERA    193
FZD3_      HS------------LFSCEPITLRMCQD--LPYNTTFMPNLLNHYDQQTAALAMEPFHPMVNLD-CSRDFRPFLCALYAPICMEYGRVTLP-CRRLCQRA    106
FZD4_      PARGFGDEEER-----RCDPIRISMCQN--LGYNVTKMPNLVGHELQTDAELQLTTFTPLIQYG-CSSQLQFFLCSVYVPMCTEKINIPIGPCGGMCLSV    124
FZD5_      RA-AAASKAP------VCQEITVPMCRG--IGYNLTHMPNQFNHDTQDEAGLEVHQFWPLVEIQ-CSPDLRFFLCSMYTPICLPDYHKPLPPCRSVCERA    112
FZD6_      HS------------LFTCEPITVPRCMK--MAYNMTFFPNLMGHYDQSIAAVEMEHFLPLANLE-CSPNIETFLCKAFVPTCIEQIHVVPP-CRKLCEKV    102
FZD8_      AA-AASAKEL------ACQEITVPLCKG--IGYNYTYMPNQFNHDTQDEAGLEVHQFWPLVEIQ-CSPDLKFFLCSMYTPICLEDYKKPLPPCRSVCERA    114
FZD9_      PERGRGAAP--------CQAVEIPMCRG--IGYNLTRMPNLLGHTSQGEAAAELAEFAPLVQYG-CHSHLRFFLCSLYAPMCTDQVSTPIPACRPMCEQA    118
FZD10_     ERPGDGK----------CQPIEIPMCKD--IGYNMTRMPNLMGHENQREAAIQLHEFAPLVEYG-CHGHLRFFLCSLYAPMCTEQVSTPIPACRVMCEQA    113
SMO_       PLSHCGRAAP-------CEPLRYNVCLGSVLPYGATSTLLAGDSDSQEEAHGKLVLWSGLRNAPRCWAVIQPLLCAVYMPKCE--NDRVELPSRTLCQAT    150
           .......110.......120.......130.......140.......150.......160.......170.......180.......190.......200
```

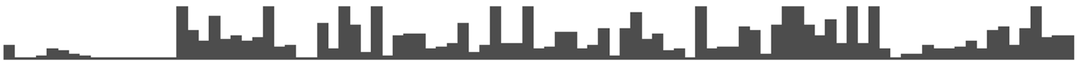

Continued

```
             *  :       **: : *
FZD7_     RQGCEALMNKFGFQWPERLRCENFPVHG--AGEICVGQNTSDGSGGPGGGPTAYPTAPY-----------LPDLP-----------------------FT   191
FZD1_     RQGCEALMNKFGFQWPDTLKCEKFPVHG--AGELCVGQNTSDKGTPTPSLLPEFWTSNP-----------QHGGG-------------------GHRGGF   262
FZD2_     RQGCEALMNKFGFQWPERLRCEHFPRHG--AEQICVGQNHSE--DGAPALLTTAPPPGL-----------QPGAG-------------------GTPGG-   259
FZD3_     YSECSKLMEMFGVPWPEDMECSRFPDCD-EPYPRLVDLNLAGEPTEGAPVAVQR----------------DYG---------------------------   163
FZD4_     KRRCEPVLKEFGFAWPESLNCSKFPPQN-DHNHMCMEGP------------GDEEVPLP----------HKT----------------------------   174
FZD5_     KAGCSPLMRQYGFAWPERMSCDRLPVLGRDAEVLCMDYNRSEATTAPPRPFPAKPTLP------------GPPGA-------------------PASGG-   181
FZD6_     YSDCKKLIDTFGIRWPEELECDRLQYCD-ETVPVTFDPHTEFLGPQKKTEQVQR----------------DIG---------------------------   159
FZD8_     KAGCAPLMRQYGFAWPDRMRCDRLPEQG-NPDTLCMDYNRTDLTTAAPSPPRRLPPPPPGEQPPSGSGHGRPPGARPPHRGGGRGGGGGDAAAPPARGGG   213
FZD9_     RLRCAPIMEQFNFGWPDSLDCARLPTRN-DPHALCMEAPENATAGPAEPHKGLGMLPVA----------PRPARP-------------------P----G   184
FZD10_    RLKCSPIMEQFNFKWPDSLDCRKLPNKN-DPNYLCMEAPNN---GSDEPTRGSGLFPPL----------FRPQRP-------------------HSAQEH   180
SMO_      RGPCAIVERER--GWPDFLRCTPDRFPEGCTNEVQNIKFNSSG---------------------------------------------------------   192
          .......210.......220.......230.......240.......250.......260.......270.......280.......290.......300
```

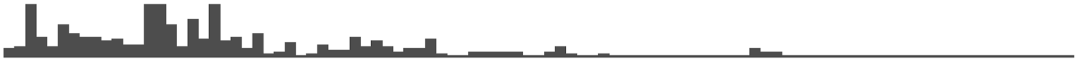

```
                                                                            ::   . .   .:  *. *:: :  .   **
FZD7_     ALPPGASDGRGRPAFPFSCPRQLKVPPYLG--YRFLGERDCGAPCEPGRANGLMYFKEEERRFARLWVGVWSVLCCASTLFTVLTYLVDMRR-FSYPERP   288
FZD1_     PGGAGASER-----GKFSCPRALKVPSYLN--YHFLGEKDCGAPCEPTKVYGLMYFGPEELRFSRTWIGIWSVLCCASTLFTVLTYLVDMRR-FSYPERP   354
FZD2_     PGGGGAPPRYATLEHPFHCPRVLKVPSYLS--YKFLGERDCAAPCEPARPDGSMFFSQEETRFARLWILTWSVLCCASTFFTVTTYLVDMQR-FRYPERP   355
FZD3_     ----------------FWCPRELKIDPDLG--------YSFLHVRDCSPPCPNMYFRREELSFARYFIGLISIICLSATLFTFLTFLIDVTR-FRYPERP   237
FZD4_     PIQPGEECHS-------VGTNSDQYIWVKR-------SLNCVLKCGYD---AGLY-SRSAKEFTDIWMAVWASLCFISTAFTVLTFLIDSSR-FSYPERP   254
FZD5_     ----ECPAGG-----PFVCKCREPFVPILKESHPLYNKVRTGQVPNCAVPCYQPSFSADERTFATFWIGLWSVLCFISTSTTVATFLIDMER-FRYPERP   270
FZD6_     ----------------FWCPRHLKTSGGQG--------YKFLGIDQCAPPCPNMYFKSDELEFAKSFIGTVSIFCLCATLFTFLTFLIDVRR-FRYPERP   233
FZD8_     GGGKARPPGGGAAPCEPGCQCRAPMVSVSSERHPLYNRVKTGQIANCALPCHNPFFSQDERAFTVFWIGLWSVLCFVSTFATVSTFLIDMER-FKYPERP   312
FZD9_     DLGPGAGGSG-------TCENPEKFQYVEK-------SRSCAPRCGPG---VEVFWSRRDKDFALVWMAVWSALCFFSTAFTVLTFLLEPHR-FQYPERP   266
FZD10_    PLKDGGPGRG-------GCDNPGKFHHVEK-------SASCAPLCTPG---VDVYWSREDKRFAVVWLAIWAVLCFFSSAFTVLTFLIDPAR-FRYPERP   262
SMO_      -----------------QCEVPLVRTDNPK--------SWYEDVEGCGIQCQNPLFTEAEHQDMHSYIAAFGAVTGLCTLFTLATFVADWRNSNRYPAVI   266
          .......310.......320.......330.......340.......350.......360.......370.......380.......390.......400
```

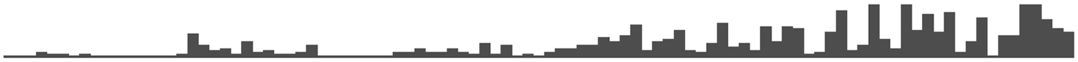

```
          :::   *:  ::   .                                                                   *.::*:::*:  **. :
FZD7_     IIFLSGCYFMVAVAHVAGFLLEDRAVCVER------------------------------------FSDDGYRTVAQGTKKEGCTILFMVLYFFGMASSI   352
FZD1_     IIFLSGCYTAVAVAYIAGFLLEDRVVCNDK------------------------------------FAEDGARTVAQGTKKEGCTILFMMLYFFSMASSI   418
FZD2_     IIFLSGCYTMVSVAYIAGFVLQERVVCNER------------------------------------FSEDGYRTVVQGTKKEGCTILFMMLYFFSMASSI   419
FZD3_     IIFYAVCYMMVSLIFFIGFLLEDRVACNAS-----------------------------------IPAQYKASTVTQGSHNKACTMLFMILYFFTMAGSV   302
FZD4_     IIFLSMCYNIYSIAYIVRLTVGRERISCDF------------------------------------EEAAEPVLIQEGLKNTGCAIIFLLMYFFGMASSI   318
FZD5_     IIFLSACYLCVSLGFLVRLVVGHASVACSR---------------------------------------EHNHIHYETTGPALCTIVFLLVYFFGMASSI   331
FZD6_     IIYYSVCYSIVSLMYFIGFLLGDSTACNKA-----------------------------------DEKLELGDTVVLGSQNKACTVLFMLLYFFTMAGTV   298
FZD8_     IIFLSACYLFVSVGYLVRLVAGHEKVACSGGAPGAGGAGGAGGAAAGAGAAGAGAGGPGGRGEYEELGAVEQHVRYETTGPALCTVVFLLVYFFGMASSI   412
FZD9_     IIFLSMCYNVYSLAFLIRAVAGAQSVACD-------------------------------------QEAGALYVIQEGLENTGCTLVFLLLYYFGMASSL   329
FZD10_    IIFLSMCYCVYSVGYLIRLFAGAESIACD-------------------------------------RDSGQLYVIQEGLESTGCTLVFLVLYYFGMASSL   325
SMO_      LFYVNACFFVGSIGWLAQFMDGARREIVCR------------------------------------ADGTMRLGEPTSNETLSCVIIFVIVYYALMAGVV   330
          .......410.......420.......430.......440.......450.......460.......470.......480.......490.......500
```

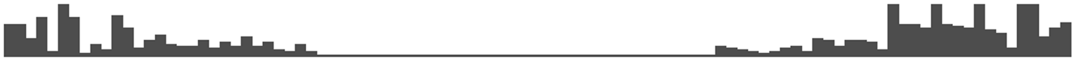

```
          *:*:*: :*. :        :.:      **  :*  *   ::  * :  : .* ::*:*:*.  .      **: *:   :.:*  ::  *.::*  ::
FZD7_     WWVILSLTWFLAAGMKWG-HEAIEANSQYFHLAAWAVPAVKTITILAMGQVDGDLLSGVCYVGLSSVDALRGFVLAPLFVYLFIGTSFLLAGFVSLFRIR   451
FZD1_     WWVILSLTWFLAAGMKWG-HEAIEANSQYFHLAAWAVPAIKTITILALGQVDGDVLSGVCFVGLNNVDALRGFVLAPLFVYLFIGTSFLLAGFVSLFRIR   517
FZD2_     WWVILSLTWFLAAGMKWG-HEAIEANSQYFHLAAWAVPAVKTITILAMGQIDGDLLSGVCFVGLNSLDPLRGFVLAPLFVYLFIGTSFLLAGFVSLFRIR   518
FZD3_     WWVILTITWFLAAVPKWG-SEAIEKKALLFHASAWGIPGTLTIILLAMNKIEGDNISGVCFVGLYDVDALRYFVLAPLCLYVVVGVSLLLAGIISLNRVR   401
FZD4_     WWVILTLTWFLAAGLKWG-HEAIEMHSSYFHIAAWAIPAVKTIVILIMRLVDADELTGLCYVGNQNLDALTGFVVAPLFTYLVIGTLFIAAGLVALFKIR   417
FZD5_     WWVILSLTWFLAAGMKWG-NEAIAGYAQYFHLAAWLIPSVKSITALALSSVDGDPVAGICYVGNQNLNSLRGFVLGPLVLYLLVGTLFLLAGFVSLFRIR   430
FZD6_     WWVILTITWFLAAGRKWS-CEAIEQKAVWFHAVAWGTPGFLTVMLLAMNKVEGDNISGVCFVGLYDLDASRYFVLLPLCLCVFVGLSLLLAGIISLNHVR   397
FZD8_     WWVILSLTWFLAAGMKWG-NEAIAGYSQYFHLAAWLVPSVKSIAVLALSSVDGDPVAGICYVGNQSLDNLRGFVLAPLVIYLFIGTMFLLAGFVSLFRIR   511
FZD9_     WWVVLTLTWFLAAGKKWG-HEAIEAHGSYFHMAAWGLPALKTIVILTLRKVAGDELTGLCYVASTDAAALTGFVLVPLSGYLVLGSSFLLTGFVALFHIR   428
FZD10_    WWVVLTLTWFLAAGKKWG-HEAIEANSSYFHLAAWAIPAVKTILILVMRRVAGDELTGVCYVGSMDVNALTGFVLIPLACYLVIGTSFILSGFVALFHIR   424
SMO_      WFVVLTYAWHTSFKALGTTYQPLSGKTSYFHLLTWSLPFVLTVAILAVAQVDGDSVSGICFVGYKNYRYRAGFVLAPIGLVLIVGGYFLIRGVMTLFSIK   430
          .......510.......520.......530.......540.......550.......560.......570.......580.......590.......600
```

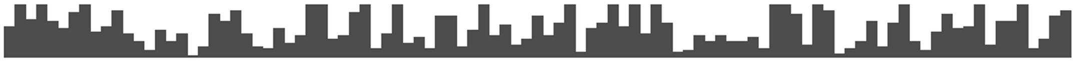

Continued

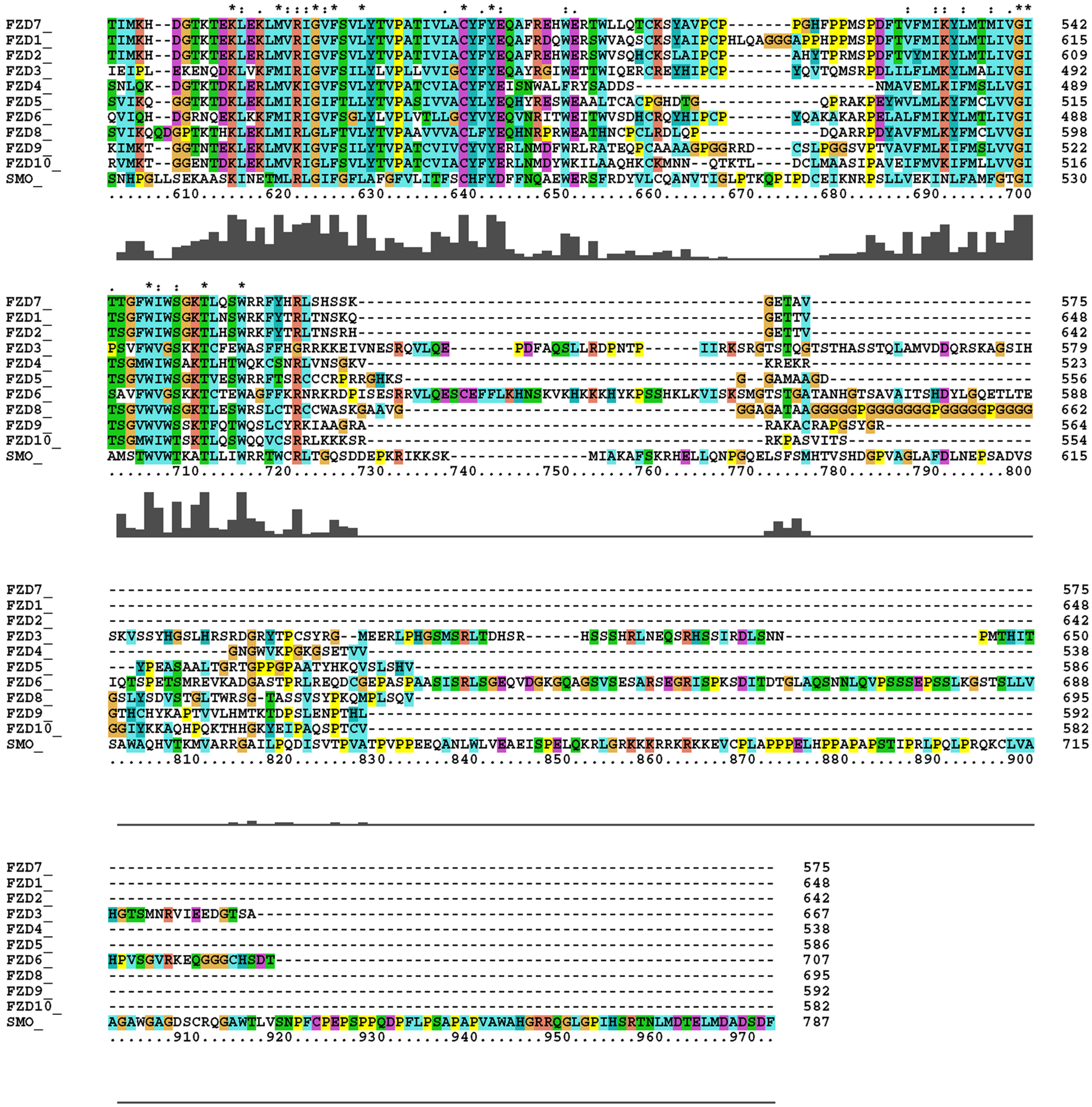

*Multiple sequence alignment result using *clustalX* program.

Supplementary Table 3: The first set of 67 SBVS-selected compounds.

See Supplementary File 2

Supplementary Table 4: The second set of 35 selected analogs.

See Supplementary File 3
